# Hope, optimism, and pessimism as predictors of positive and negative psychological changes related to the COVID-19 pandemic in Slovak adults

**DOI:** 10.3389/fpsyg.2023.1151027

**Published:** 2023-07-28

**Authors:** Erika Jurišová, Lucia Pivková, Lucia Ráczová, Tomáš Sollár, Martina Romanová

**Affiliations:** ^1^Department of Psychological Sciences, Faculty of Social Sciences and Health Care, Constantine the Philosopher University in Nitra, Nitra, Slovakia; ^2^Institute of Applied Psychology, Faculty of Social Sciences and Health Care, Constantine the Philosopher University in Nitra, Nitra, Slovakia

**Keywords:** hope, optimism, pessimism, positive and negative psychological changes in outlook, COVID-19

## Abstract

**Background and objectives:**

Positive and negative changes in outlook represent psychological changes that are the results of the cognitive processing of stressful and traumatic events by an individual. The objectives of the study were (1) to determine the level of occurrence and types of positive and negative changes in connection with the COVID-19 pandemic among adults in Slovakia and (2) to study the role of personality factors such as hope (dispositional and perceived) and life orientation (optimism and pessimism) in the prediction of positive and negative changes in adults during the fourth pandemic wave.

**Methods:**

A Short Form of the Changes in Outlook Questionnaire (CiOQ-S), the Dispositional Hope Scale (DHS), the Perceived Hope Scale (PHS), and the Life Orientation Test (LOT-R) were administered. The research sample consisted of 102 participants, whose ages ranged from 20 to 65 years (*M*_*age*_ = 38.90, *SD* = 14.28). The research design was quantitative, exploratory, and confirmatory.

**Results:**

In total, 95% of participants reported positive changes related to COVID-19. Concurrently, up to 70% of these participants also reported negative changes from the impact of the pandemic. Only 25% of participants reported positive changes without noticing any negative perception of the consequences of the pandemic. Overall, 68% of participants reported negative changes related to COVID-19. Only 29% of participants reported negative changes without noticing any positive perception of the consequences of the pandemic. In total, up to 86% of participants agreed with experienced psychological changes (positive or negative) as a result of the COVID-19 pandemic. The high prevalence of positive changes along with the relatively high prevalence of negative changes related to the COVID-19 pandemic outline the question of whether reported positive changes represent real or illusory growth. Optimism and pessimism were found to be significant independent predictors of positive changes related to the COVID-19 pandemic. Hope was identified as a significant independent predictor of negative changes related to the COVID-19 pandemic.

## Introduction

The novel coronavirus (COVID-19) emerged in Wuhan province in China in December 2019. In the period of the following few months, the virus spread around the world and caused a worldwide pandemic. By 31 December 2020, there were more than 82 million confirmed cases worldwide, with more than 1.8 million deaths (World Health Organization, 2021). The first confirmed case in Slovakia was detected on 6 March 2020 (Ministry of Health of the Slovak Republic, 2020). At the time of research data collection in February and March 2022, the global COVID-19 pandemic recorded 441,045,255 cases of infection and 6,013,580 deaths since its outbreak. In Slovakia, during this period, 1,458,129 cases of infection among the ~5,428,792 inhabitants and 18,530 deaths were recorded (source https://www.worldometers.info/coronavirus).

Acute and chronic negative psychological, mental, and emotional consequences of the COVID-19 pandemic have been documented in many studies worldwide [e.g., higher levels of stress, anxiety, depression, frustration, post-traumatic symptoms, insomnia, higher level of fear of infection, and loneliness (Arora and Grey, 2020; Cénat et al., 2020; Mazza et al., 2020; Rajkumar, 2020; Xiong et al., 2020; Zhou et al., 2020 and others)]. Currently, there is an increasing number of studies that focus on describing and providing deeper understanding of positive psychological changes (e.g., in the context of post-traumatic growth) as a possible consequence of people's experience with the COVID-19 pandemic (Britton et al., 2019; Tamiolaki and Kalaitzaki, 2020; Asmundson et al., 2021; Lau et al., 2021; Park and Im, 2021; Gökalp et al., 2022). Systematic reviews (e.g., Manchia et al., 2022) summarizing the impact of the COVID-19 pandemic on stress resilience and mental health have found that the effects of the pandemic, whether related to COVID-19 itself or related measures, are surprisingly heterogeneous across populations. *Therefore, the first aim of this study was to determine the level of occurrence and types of positive and negative psychological changes in connection with the COVID-19 pandemic among adults in Slovakia*.

Another significant finding from the study conducted by Manchia et al. (2022) was that the effects of stress and resilience capacity rely on various (neuro)biological, psychological, and environmental factors. Furthermore, these effects are highly influenced by an individual's unique circumstances. Stueck (2021) presented a comprehensive theory, known as the Pandemic Management Theory (PMT), to explain the psychological mechanisms underlying coping with and processing a pandemic. The PMT is based on a biocentric health management approach. According to the PMT, being healthy during a pandemic entails maintaining a congruent connection between the biological and psychological levels and preserving the fundamental mechanisms of autoregulation and autopoiesis in the biocentric core (Stueck, 2021). One of the theses of the PMT states that people's identity is threatened by more fears than just the fear of death alone. These types of fear include the fear of contracting and falling ill and the fear of losing autonomy. These fears can impact self-esteem, cultural values, physical experience, and ultimately affect hormonal and central nervous processes, as well as the stability of the immune system in the biocentric core (Goldenberg et al., 2000). Stueck (2021) defined six phases of coping with the burden of the lockdown and the further load process of the COVID-19 pandemic. The first phase involves interpreting the pandemic situation with its load. This phase includes two evaluative processes: situation and reaction-oriented evaluation and the assessment of coping resources. The assessment processes are influenced by situational and habitual factors, such as personality aspects (internal–external orientation, anxiety and cognitive styles, risk attitude, and overall personality) and behavior. Positive experiences in managing and interpreting the burden (e.g., viewing challenges, having faith in one's coping resources, and self-belief) during the pandemic can lead to positive changes in later phases. *Therefore, the second aim of the research was to study the role of personality factors such as hope (dispositional and perceived) and life orientation (optimism and pessimism) in the prediction of positive and negative changes in adults during the COVID-19 pandemic*. We perceive the identification of specific personality factors causing different resistance and vulnerability to the pandemic as important information for defining effective therapeutic strategies and developing an effective approach to public health.

## Positive and negative psychological changes as a response to stressful events

Joseph et al. (2005) assume that negative and positive psychological changes occur when people are confronted with stressful/traumatic events (directly or indirectly) which challenge their assumptions about themselves and the world. The organismic valuing theory of growth through adversity by Joseph and Linley (2005, p. 1) “*posits an intrinsic motivation toward growth, showing how this leads to the states of intrusion and avoidance that are characteristic of cognitive-emotional processing after trauma”*. The theory posits three possible outcomes of this cognitive-emotional processing: assimilation, negative accommodation (negative personality changes, depression, and learned helplessness), and positive accommodation (positive changes and personality growth). The positive changes following adversity can be manifested in three basic areas: perception of self, philosophy of life, and relationships. The theory describes personal changes as gaining wisdom, acquiring inner strength, or a greater ability to sympathize with others. The theory also understands a change in life philosophy as a change in life values. A change in relationships is characterized by the fact that people adopt new attitudes toward their closest ones. Due to the trauma people have experienced, they value their family and friends more (Joseph, 2017).

Researchers have used various alternative terms to denote positive changes that appear as a result of the struggle with adversity and lead the individual to achieve a higher level of functioning than before the event. One of the most elaborated concepts is “*post-traumatic growth”* (PTG). Tedeschi and Calhoun defined PTG as the phenomenon of “*positive psychological change experienced as a result of the struggle with highly challenging life circumstances”* (Tedeschi and Calhoun, 2004, p. 1). In the functional-descriptive model, PTG is defined as a significant positive change in the individual's cognitive and emotional life, which can have its external manifestations in behavior (Tedeschi and Calhoun, 2004).

The pandemic has incurred significant psychological stress among those affected (Song, 2020). Nevertheless, recently, an increasing number of studies have reported high percentages of positive changes and psychological growth related to the COVID-19 pandemic. For example, Asmundson et al. (2021) conducted an analysis of studies monitoring the prevalence of PTG due to the pandemic and found that 39–89% of participants reported PTG. They also found that ~77% of participants reported growth, the most common being developing a greater appreciation for healthcare workers, for the value of one's own life, for friends and family, for each day, and changing priorities about what is important in life and greater feelings of self-reliance. In addition, later published studies, e.g., Xie and Kim (2022), claim that more than half of the participants (60.8%) reported PTG during the pandemic. To clarify the prevalence of growth related to the COVID-19 pandemic, a partial objective was added to the first one, and we decided to determine the level of occurrence of positive and negative changes in connection to the COVID-19 pandemic among adults in Slovakia. Recent evidence indicates that it is important to distinguish between real and illusory PTG. New findings suggest that some self-reported PTG may reflect dysfunctional coping strategies for stressful events, but people may report experiencing PTG as part of a self-deceptive strategy. By implementing this strategy, people try to persuade themselves that they cope with the situation better than they do (Asmundson et al., 2021). In alignment with the above-mentioned, the Short Form of Changes in Outlook (CiOQ-S) developed by Joseph et al. (1993) was administered to the participants. The advantage of this tool is that it measures positive and negative reactions to stressful and traumatic events, thus offering a more comprehensive picture of ongoing changes (Joseph et al., 2005). The CiOQ-S is designed to gauge the outcomes of cognitive processing of traumatic events as demonstrated by subjectively observed psychological changes (Ba, 2007). Joseph et al. (2006) in their validation studies found positive correlations between negative psychological changes and symptoms of post-traumatic stress disorder; on the contrary, positive psychological changes were significantly related to post-traumatic growth.

## Hope, optimism, and pessimism

Snyder (2002) conceptualized hope as a trait-like cognitive construct encompassing affirmative positive beliefs about one's ability to accomplish personal goals. Dispositional hope has been conceptualized as consisting of two constructs: pathways and agency. The pathways component reflects an individual's perceived means or routes available to achieve goals. The agency is described as the belief in one's ability to succeed in using pathways to achieve desired aims and is characterized by determination, motivation, and energy directed toward meeting one's goals (Creamer et al., 2019). Thus, Snyder's Hope Theory also includes goals (Snyder, 2002) as mental targets that anchor agency and pathways. People with high hope have more resources to overcome their difficulties, have the ability to generate possible means, identify multiple viable routes of attaining desired goals, find alternative routes when their initial strategies fail, and have greater confidence in applying these coping strategies (Creamer et al., 2019).

Apart from the concept of dispositional hope, there is also a new, independent concept of perceived hope that was postulated by Krafft et al. (2017), and that differs from Snyder's dispositional hope. Perceived hope is understood as a deep trust in the positive development of the event, especially in difficult life situations beyond our control. Krafft et al. (2017) tried to fill in the missing dimensions in Snyder's theory of hope (spiritual and relational level) and added aspects of human destiny that a person cannot influence. Perceived hope may flow from self-transcendent sources and is strongly associated with needs such as experiencing the meaning of life, helping others, developing close and intimate relationships, and spiritual and religious experiences (Krafft and Walker, 2018; Slezáčková et al., 2020).

There is a growing body of evidence supporting the notion that hope functions to drive adaptive behavior (Folkman, 2013). Fredrickson (2001) states that hope has the potential to influence people to adjust their relationship with negative thoughts and emotions by focusing on positivity and that improves their ability to cope with stressful life events. Several studies from the pandemic period point to findings that hope had a direct effect on the improvement of psychological health and wellbeing during the early stage of the COVID-19 pandemic and that adults with high hope are more likely to be capable of bouncing back from stressful situations and have greater subjective wellbeing and better psychological health (Yildirim and Arslan, 2020). Hope was found to be associated with reduced pandemic stress and increased wellbeing by serving as an adaptive mechanism for recovering from stress (Gallagher et al., 2021).

In addition to hope, we also decided to explore life orientation (optimism and pessimism) as a personality factor that might potentially predict positive and negative psychological changes related to the COVID-19 pandemic. Emerging empirical evidence suggests that disposition hope (Snyder, 2002) and disposition optimism (Scheier and Carver, 1985) represent conceptually related yet still distinct constructs. According to Laranjeira and Querido (2022), both concepts share several common elements: personality traits, cognitive constructs, reference to general expectancies, relation to significant personal goals, future orientation [expecting good or positive things and a better future (Bailis and Chipperfield, 2012)], and acting as determinants of behavior (Krafft et al., 2021). According to Bruininks and Malle (2005), perceived hope and optimism are two different constructs. The authors suggest that the effect of optimism is context-independent. Dispositional hope manifests itself mainly in situations that are more personally relevant and are associated with specific goals. The perspective of hope and optimism are two different constructs presented in several other studies (Yang et al., 2014; Rand, 2018; Wider et al., 2022). The results of these studies suggest that hope and optimism can predict various mental health-related consequences.

Dispositional optimism and pessimism are described as a generalized tendency to expect favorable experiences about future events (Scheier and Carver, 1985). Optimism and pessimism are conceptualized as important constructs in coping with uncontrollable life events (Nes, 2016). Seligman (1991) has applied optimism and pessimism to the ways in which people explain events in their lives. Optimism and pessimism are stable personality characteristics that have important implications for regulating one's behavior (Arslan et al., 2021). The issue of whether optimism and pessimism are two ends of independent continuums or represent two opposite ends of the same continuum has not been resolved yet. We are inclined to believe that these two constructs represent two unipolar dimensions, that is, the opposite of optimism is the lack of optimism, which is distinguishable from the presence of pessimism (Marshall et al., 1992).

Optimism increases people's motivation to pursue goal-oriented behavior (Scheier and Carver, 1985). Optimistic individuals use more problem-focused strategies (adaptive coping strategies) that contribute to better adaptation and proactive functioning when facing negative life events compared with those who are pessimistic (Nes and Segerstrom, 2006; Nes, 2016). Recent studies revealed that optimism helped to protect mental health, lessen psychological distress, and lower anxiety and depression (Carver and Scheier, 2014; Kwok and Gu, 2017; Fischer et al., 2018). A meta-analytic review by Bostock et al. (2009) demonstrates that the relationships between PTG, optimism, and pessimism are ambiguous. At the same time, little is known about the role of pessimism in relation to post-traumatic changes, whereas optimism was more frequently studied in relation to PTG. Recent studies (Laslo-Roth et al., 2020; Koliouli and Canellopoulos, 2021; Di Corrado et al., 2022) confirmed that a prominent level of hope and optimism during the pandemic period could be a significant factor in attaining goals. Moreover, hope and optimism can help people reframe a traumatic event in a positive perspective that highlights opportunities for personal growth. The cited studies did not examine pessimism in relation to PTG. A study conducted by Britton et al. (2019) examined both factors (optimism and pessimism) and found that optimism was a predictor of PTG, and pessimism did not appear to influence PTG. Arslan et al. (2021) found that higher optimism and lower pessimism can reduce the negative impact of psychological inflexibility on the experience of psychological problems, and optimism partially mediated the relationship between coping flexibility and both psychological problems and wellbeing during the COVID-19 pandemic.

Based on the above review, the second aim of our study was to determine the role of hope and life orientation in predicting positive and negative psychological changes related to COVID-19 in adults using regression models. In the first model for positive changes, we assumed that hope (dispositional and perceived) and optimism will increase the frequency of positive psychological changes related to the COVID-19 pandemic, and conversely, pessimism will decrease the frequency of positive changes. In the second model for negative psychological changes, we assumed that hope (dispositional and perceived) and optimism will decrease the frequency of negative reactions, and conversely, pessimism will increase the frequency of negative changes.

## Method

### Sample and procedure

The research sample consisted of 102 participants aged between 20 and 65 years (43 men and 59 women, *M*_*age*_ = 38.90, *SD* = 14.28). Data were collected from February 2022 to March 2022 using the snowball sampling technique, and we used pen-and-paper and online methods for data collection. This approach to data collection was chosen due to restrictions resulting from public health measures during the COVID-19 pandemic. It took ~20 min to complete the questionnaires.

### Materials

*The Dispositional Hope Scale* (DHS; Snyder et al., 1991) is a 12-item self-report scale that assesses the sense of hope. Four items measure the agency factor, e.g., “*I energetically pursue my goals.”*, and four items measure the pathway factor, e.g., “*I can think of many ways to get the things in life that are important to me.”* Four items are used as distractors, e.g., “*I feel tired most of the time.”* The items are scored on a 4-point scale ranging from “*absolutely false”* to “*absolutely true.”* The score range is 4–16 on each scale and 8–32 on the total scale.

*The Perceived Hope Scale* (PHS; Krafft et al., 2017) is a unidimensional scale that includes six items, such as “*Hope outweighs anxiety in my life”*. The questions are rated on a 6-point Likert scale from 0 “*strongly disagree”* to 5 “*strongly agree”*. The total score range is 6–30.

*The Revised Life Orientation Test* (LOT-R; Scheier et al., 1994) is used to measure the level of dispositional optimism as predisposition expectations of positive outcomes. It is composed of 10 items, in which subjects indicate the degree of agreement or disagreement with statements such as “*Overall, I expect more good things to happen to me than bad.”*. It uses a scale of 5 points, where 0 corresponds to “*strongly disagree”* and 4 corresponds to “*strongly agree”*. Three items are positively formulated (optimistic), three are negatively formulated (pessimistic), and four items are used for control. The score range is 3–15 on each scale. With regard to the interpretation of the test, there are two options (Ferrando et al., 2002): on the one hand, each factor can be measured separately, the option we adopted; on the other hand, total optimism can be measured by reversing the scores of the items drafted with a negative direction.

*The Short Form of Changes in Outlook* (CiOQ-S; Joseph et al., 2006) is a self-report instrument designed to measure positive and negative psychological changes following the experience of stressful events. The CiOQ-S is a 10-item instrument consisting of five items measuring positive changes (CiOP-S: e.g., “*I value my relationships much more now.”*) and 5 items measuring negative changes (CiON-S: e.g., “*My life has no meaning anymore.”*). In line with the purpose of the research, the participants were asked to answer all questions related to positive and negative changes, specifically in association with the pandemic, and to focus on those changes that they could identify in their lives. Each item is rated on a 6-point scale from strongly disagree (1) to strongly agree (6) so that there is a potential range of scores of 5–30 for both CiOP-S and CiON-S. Higher scores indicate greater positive and negative psychological changes.

### Data analysis

Statistical analyses were performed using IBM SPSS Statistics 24.0. Descriptive statistics (mean, SD) were used to describe the basic features of the dataset. Skewness and kurtosis of all variables were examined with all values in the range from −1.04 to 1.21. When transformed to *z*-scores (Field, 2009), values in absolute value were not >1.96. Therefore, parametric tests (paired Student's *t*-test, and the Pearson product-moment correlation coefficient) were used. To assess the strength of the relationship and the effect size, Cohen's *d* was used with the following interpretation: the results of interval 0.1–0.3 indicate a small relationship/difference; 0.3–0.5 indicate a medium relationship/difference; and 0.5 and above indicate a large relationship/difference. Multiple linear regression analysis (Enter method) was used for the analysis of models for predicting the positive and negative changes. We tested data for linear regression assumptions, especially multicollinearity and independence of residuals. Analysis of collinearity statistics shows that this assumption has been met, as Variance Inflation Factor (VIF) scores were below 10 and tolerance scores above 0.2 [VIF_(max)_ = 2.67 and Tolerance_(min)_ = 0.37, respectively]. The Durbin–Watson statistic (independence of residuals) showed that this assumption had been met for both DVs, as the obtained value was close to 2 (Durbin–Watson = 1.90, resp. 2.06).

## Results

### Prevalence of positive and negative psychological changes related to the COVID-19 pandemic

Perceived positive changes in all participants reached a minimum value of 8 and a maximum value of 30, with an average value of 21.57. Negative changes ranged from 5 to 30 points, with an average value of 12.85, which indicates a higher rate of positively perceived changes in the Slovak research sample. The difference is large and statistically significant [*M_*_*CiOP*−*S*_ = 21.57; SD = 4.96 vs. *M_*_*CiON*−*S*_ = 12.85; *SD* = 5.32; *t*_(101)_ = 13.049; *p* = 0.000].

Up to 95% of participants in the CiOP-S subscale reported moderate-to-strong COVID-related growth, specifically in respect to at least one positive change. Additionally, up to 70% of participants in our research group expressed moderate-to-strong agreement with statements that highlighted experiencing negative changes associated with the COVID-19 pandemic, also in at least one aspect. Based on this significant finding, we can conclude that only 25% of participants reported moderate-to-strong COVID-related growth, at least in one aspect of a positive change, without concurrent mild to strong agreement with statements focused on experiencing negative changes in connection with the COVID-19 pandemic. In total, 68% of participants indicated moderate-to-strong agreement with statements focused on experiencing negative changes in connection with the COVID-19 pandemic in at least one aspect, in the CiON-S subscale. Only 29% of participants reported moderate-to-strong COVID-related negative changes, at least in one aspect, without concurrent agreement (mild to strong) with statements focused on experiencing positive changes in connection with the COVID-19 pandemic. In total, up to 86% of participants agreed with experiencing psychological changes (positive or negative) as a result of the COVID-19 pandemic, in respect to at least one change. Only 16% of participants non-reported moderate-to-strong COVID-related positive and negative changes.

When performing the CiOQ-S item analysis, we were inspired by the analysis carried out by Asmundson et al. (2021). Item scores on the CiOQ-S along with the percentage of participants reporting moderate-to-high COVID-related positive and negative psychological changes on each item (scoring ≥ 4 on each item) are presented in Table 1. Up to 91% of participants reported a change in social relationships in connection with the COVID-19 pandemic (Item 4 “*I value my relationships much more now.”*). Similarly, 91% of participants reported a personal change associated with gaining wisdom (Item 6: “*I no longer take things or people for granted.”*). The least common type of growth was related to Item 3: “*I don't take life for granted anymore.”* The results for negative changes related to COVID-19 show that up to 90% of participants reported a loss of trust in people (Item 7). The least common type of negative change was a loss of meaning in life (12% of participants) (Item 2).

**Table 1:** Item scores on the CiOQ-S and percentage of respondents reporting moderate-to-high COVID-19 pandemic-related positive and negative psychological changes.

**Subscale**	**Item**	***M (SD)***	**% reporting moderate-to-high changes in outlook**
Negative changes (CiOQ-S: CiON-S)	1. I don't look forward to the future anymore.	2.02 (1.23)	24.4
Negative changes (CiOQ-S: CiON-S)	2. My life has no meaning anymore.	1.61 (1.17)	12.7
Positive changes (CiOQ-S: CiOP-S)	3. I don't take life for granted anymore.	3.65 (1.63)	72.5
Positive changes (CiOQ-S: CiOP-S)	4. I value my relationships much more now.	4.62 (1.26)	91.3
Positive changes (CiOQ-S: CiOP-S)	5. I am a more tolerant and understanding person now.	4.37 (1.34)	86.3
Positive changes (CiOQ-S: CiOP-S)	6. I no longer take things or people for granted.	4.67 (1.25)	91.2
Negative changes (CiOQ-S: CiON-S)	7. I Have very little trust in other people now.	3.71 (1.54)	90.5
Negative changes (CiOQ-S: CiON-S)	8. I feel very much as if I am in limbo.	2.82 (1.03)	56.8
Negative changes (CiOQ-S: CiON-S)	9. I have very little trust in myself now.	2.70 (1.48)	46.1
Positive changes (CiOQ-S: CiOP-S)	10. I value other people more now.	4.27 (1.23)	89.3

CiOQ-S: CiOP-S, The Short Form of Changes in Outlook: Positive changes; CiOQ-S: CiON-S, The Short Form of Changes in Outlook: Negative changes; *M*, mean; *SD*, standard deviation.

### Correlation analysis

A correlational matrix in Table 2 points to the relationships between personal variables such as hope (dispositional and perceived), optimism, pessimism, and positive and negative changes in the participants during the COVID-19 pandemic. Positive changes were positively related to perceived hope and optimism, and the strength of the relationships varied from small to moderately strong (according to Cohen's *d*). We did not find statistically significant relationships between positive changes, pessimism, and dispositional hope. On the contrary, in the case of negative changes, we found statistically significant negative relationships with all variables, such as dispositional hope (agency and pathways), perceived hope, and optimism. The values of the correlation coefficient varied from medium to large. Negative changes were positively and moderately correlated with pessimism.

**Table 2:** Correlation matrix (*r*) with Pearson's correlation coefficients and 95% confidence intervals, reliability.

	**1. DHS (pathways)**	**2. DHS (agency)**	**3. PHS (perceived hope)**	**4. LOT-R (optimism)**	**5. LOT-R (pessimism)**	**6. CiOQ-S (positive changes)**	**M ±SD (95% CI of mean)**	**Reliability—Cronbach's α/McDonald's ω (number of items)**
Dispositional hope_ _Pathways_ (DHS)	–	–	–	–	–	–	12.294 ± 2.159_(11.869, 12.718)	0.741/0.752_(4)
Dispositional hope__Agency_ (DHS)	0.663^**^_(0.537, 0.759)	–					11.735 ± 1.964 (11.349, 12.121)	0.602/629 (4)
Perceived hope (PHS)	0.578^***^_(0.432, 0.694)	0.511^***^_(0.352, 0.642)					21.117 ± 5.972 (19.944, 22.290)	0.911/0.914_(6)
Optimism (LOT-R)	0.419^***^_(0.244, 0.567)	0.388^***^_(0.209, 0.541)	0.679^***^_(0.559, 0.772)	–			10.058 ± 2.957 (9.477, 10.639)	0.786/0.789 (3)
Pessimism (LOT-R)	−0.354^***^_(−0.513, −0.171)	−0.456^***^_(−0.598, −0.287)	−0.623^***^_(−0.729, −0.488)	−0.564^***^_(−0.684, −0.415)	–		8.676 ± 2.670 (8.151, 9.201)	0.730/0.745 (3)
Positive changes (CiOQ-S: CiOP-S)	0.041 (−0.155, 0.234)	0.041 (−0.155, 0.234)	0.237^*^_(0.045, 0.412)	0.354^***^_(0.171, 0.513)	−0.022 (−0.216, 0.173)	–	21.528 ± 4.968 (20.602, 22.554)	0.786/0.851 (5)
Negative changes (CiOQ-S: CiON-S)	−0.544^***^_(−0.668, −0.391)	−0.445^***^_(−0.588, −0.274)	−0.631^***^_(−0.735, −0.498)	−0.405^***^_(−0.556, −0.228)	0.501^***^_(0.340, 0.634)	0.14(−0.056, 0.326)	12.852 ± 5.323 (11.807, 13.898)	0.843/0.817 (5)

M, mean; SD, standard deviation; *r*, Pearson's product-moment correlation coefficient; DHS, The Dispositional Hope Scale; PHS, Perceived Hope Scale; LOT-R, The Revised Life Orientation Test; CiOQ-S, The Short Form of Changes in Outlook.

*p* < 0.05^*^, *p* < 0.01^**^, *p* < 0.001^***^.

Correlations between optimism and hope (dispositional—agency, pathways, and perceived) were positive. The value of the correlation coefficient varied from medium to large. Similarly, pessimism correlated negatively with hope variables. The values of the correlation coefficient varied from medium to large.

### Linear regression analysis

The second aim of our study was to determine the role of hope (dispositional: 1. agency, 2. pathways, and 3. perceived) and life orientation (4. optimism and 5. pessimism) in predicting positive and negative changes related to COVID-19 in adults using regression models.

Positive changes were significantly explained by two out of five independent predictors (*F* = 4.71, *p* = 0.001; the overall proportion of explained variance was 19.7%). Both optimism and pessimism predicted positive changes in outlook (optimism: β = 0.448, pessimism: β = 0.312).

Negative changes were statistically significantly explained by two independent predictors out of five (*F* = 17.18, *p* < 0.000; the overall proportion of explained variance was 47.2%). Dispositional hope_ _Pathways_ and perceived hope predicted negative changes in outlook (dispositional hope_ _Pathways_: β = −0.278, perceived hope: β = −0.420) (Table 3).

**Table 3:** Regression models for prediction of positive and negative psychological changes related to the COVID-19 pandemic (CiOQ-S).

	***R^2^***	***F***	***B***	***SE (B)***	***B***	***t***
**Positive changes (CiOQ-S)**	0.197	4.71^**^				
Dispositional hope__Pathways_ (DHS)			−0.389	0.306	−0.169	−1.27
Dispositional hope__Agency_ (DHS)			0.024	0.326	0.009	0.07
Perceived hope (PHS)			0.183	0.124	0.220	1.47
Optimism (LOT-R)			0.752	0.216	0.448	3.47^**^
Pessimism (LOT-R)			0.580	0.231	0.312	2.50^*^
**Negative changes (CiOQ-S)**	0.427	17.188^***^				
Dispositional hope__Pathways_ (DHS)			−0.686	0.266	−0.278	−2.58^**^
Dispositional hope__Agency_ (DHS)			0.014	0.283	0.005	0.051
Perceived hope (PHS)			−0.374	0.108	−0.420	−3.46^**^
Optimism (LOT-R)			0.200	0.188	0.111	1.06
Pessimism (LOT-R)			0.412	0.201	0.206	2.04

*R*^2^, coefficient of determination; *F*, Fisher's F-test; *B*, unstandardized regression coefficient; *SE(B)*, standard error of unstandardized regression coefficient; β, standardized regression coefficient; *t, t*-statistic.

*p* < 0.05^*^, *p* < 0.01^**^, *p* < 0.001^***^.

## Discussion

The first aim of this study was to determine the level of occurrence and types of positive and negative psychological changes in connection with the COVID-19 pandemic among adults in Slovakia. Data were collected from February 2022 to March 2022. During this period, the fourth pandemic wave with the dominance of the Omicron variant was on the rise. Linley and Joseph (2004) claim that growth should be evaluated as the process can take months or even years after the negative event. The way of thinking changes little by little and shifts from negative to positive thinking (Hallam and Morris, 2014). We assumed that it would be possible to monitor the development of potential positive psychological changes in individuals after 2 years of the pandemic outbreak.

Up to 95% of our participants reported moderate-to-high COVID-19-related positive psychological changes in at least one aspect. Only 25% of participants reported growth without concurrent mild to strong agreement with statements focused on experiencing negative changes in connection with the COVID-19 pandemic. Our data on the prevalence of COVID-19-related growth are lower compared to the results of foreign studies in this area, where the prevalence of PTG ranged from 39 to 89% (Asmundson et al., 2021). These differences can be explained by a different methodology for evaluating the occurrence of growth. In our study, from the participants who reported the occurrence of positive changes, we filtered out those who concurrently reported the occurrence of negative changes caused by the COVID-19 pandemic. Using this procedure, we identified the number of participants who reported growth. In studies presenting a higher prevalence of growth (Asmundson et al., 2021), data on the occurrence of PTG were reported directly, based on the participants' statements, without the specific correction that we implemented in our study.

As it was mentioned before when conducting a more detailed analysis, we found that 95% of participants reported growth related to COVID-19, and concurrently, up to 70% of them also reported negative changes. This result supports the existence of two types of growth: real and illusory, introduced by Zoellner and Maercker (2006) in the theory of the two-component model of PTG. In this model, one aspect of growth is real, functional, constructive, and self-transcending. The second component is a distorting and self-deceiving illusion. According to Taylor and Brown (1988), people who demonstrate distorting and self-deceiving illusions are typically those who experienced unrealistic optimism and a sense of control to cope with the trauma.

Similarly, Asmundson et al. (2021) identified two clusters of COVID-19-related growth in their study: real and illusory. These two clusters, as the authors' state, did not differ significantly in terms of socially desirable reaction tendencies, and the authors hypothesize that “*people with delusional growth are unlikely to be deliberately deceptive or engage in some kind of impression management”*. Our result on experiencing growth and, at the same time, experiencing negative psychological changes in connection with the COVID-19 pandemic might seem paradoxical, but it offers another direction of interpretation. In line with second-wave positive psychology, particularly with the approach taken by Wong et al. (2021), it is possible to think about our result in terms of the coexistence of positive and negative emotions and experiences. Wong et al. (2021) in the self-transcendence approach to global wellbeing states that “*every positive or negative emotion contains a seed of its opposite and therefore, it is difficult, if not impossible, to achieve flourishing without going through the gates of overcoming adversity”*. Based on this theory, we can assume that “*a mind that is big enough to hold two opposing ideas”* can help people experience PTG.

More research is needed to determine how the reported growth (whether real or illusory) in a certain phase of the COVID-19 pandemic affects the trajectory of further adaptation to its development. We are inclined to agree that illusory growth does not have to be perceived negatively; this aspect of growth can eliminate the negative effects of a traumatic event, and thus temporarily fulfill the role of another positive adaptive strategy (Mareš, 2012).

In addition to monitoring the level of occurrence of positive and negative psychological changes in connection with the COVID-19 pandemic, we also explored the areas mostly affected by these changes. Up to 91% of participants reported changes in social relationships, including higher awareness of the value of social relationships. Similarly, 91% of participants reported personal changes, specifically gaining wisdom and realizing the importance of genuine relationships. A small number of participants reported growth in terms of different perceptions of life. PTG was originally used to describe major changes in beliefs, attitudes, or ways of relating to the world, such as finding greater meaning or purpose in life, and later expanded its definition to include smaller but potentially important changes, such as an appreciation for the little things in life (Asmundson et al., 2021). Our participants, in the actual phase of the pandemic, reported COVID-19-related growth mainly in minor changes (primarily in the area of social relations), and minor changes were identified in areas of deeper, existential issues related to the meaning of life. These results seem to be obvious since PTG is a process that develops over time (Linley and Joseph, 2004). However, we are aware that such a fundamental change is very individual and complex. As for experiencing negative changes, we found that 68% of participants indicated moderate-to-strong agreement with statements focused on experiencing negative changes in connection with the COVID-19 pandemic in at least one aspect. Only 29% of participants reported negative changes without noticing any positive perception of the consequences of the pandemic. The most common negative change related to COVID-19 was a loss of trust in people, which was reported by 90% of participants. Only 12% of participants reported a loss of meaning in life related to COVID-19. No notable inclinations toward alterations in the perception of life's purpose were observed in the realm of personal development and negative transformations, even 2 years after the pandemic began. At the end of the discussion regarding the prevalence of the consequences of the COVID-19 pandemic, it is necessary to underscore that the pandemic has affected many people. This is also supported by our result that up to 86% of participants reported experiencing psychological changes (positive or negative) as a result of the COVID-19 pandemic.

The second aim of our study was to determine the role of hope and life orientation in predicting positive and negative psychological changes related to COVID-19 in adults. Life orientation (optimism and pessimism) was found to be a significant positive predictor of positive consequences of the COVID-19 pandemic. We found a positive connection between optimism and positive psychological changes which has also been confirmed by other authors (Taku and Cann, 2014; Britton et al., 2019). The result is also consistent with the idea by Britton et al. (2019) that people who report more positive emotions and personality traits show higher ego-resilience (Fredrickson and Joiner, 2002), which enables people to adapt to their environment (Klohnen, 1996) and thrive after trauma (Fredrickson et al., 2003). The effect of optimism is context-independent (Bruininks and Malle, 2005), and optimistic people have a positive outlook, believe good things will happen in the future, and are motivated to show effort even in the face of difficulties (Scheier and Carver, 1985). The abovementioned characteristics of optimism can account for people's ability to experience positive changes during the COVID-19 pandemic; therefore, despite the waves and development of the pandemic, optimistic people were able to resist and see the opportunity for growth. Genc and Arslan (2021) state that optimism provides an adaptive way to cope with stressful life events during the pandemic, which they explain by “*the adaptive role of optimism, which can be considered as a fundamental component of the ability to cope with stress experiences because it involves a positive outlook on life and that motivates individuals to undertake actions even in difficulties, and subsequently, optimism evokes favorable feelings and positivity about the future, and that may lessen the negative effects of coronavirus stress on subjective well-being”*.

However, at the same time, our findings indicated that not only the presence of optimism but also pessimism can contribute to positive psychological changes. Taku and Cann (2014) and Britton et al. (2019) did not find an effect of pessimism on PTG. Similarly, Arslan et al. (2021) found that higher optimism and lower pessimism can reduce the negative impact of psychological inflexibility on the experience of psychological problems during the COVID-19 pandemic. Our result regarding the relationship between pessimism and positive psychological changes as a response to stressful events requires further investigation. Joseph et al. (2005) state that also those factors, which are primarily perceived as “*negative”* (e.g., ruminative forms of intrusive thinking), can predict subsequent adjustment and growth. Ruminative forms of intrusive thinking support the cognitive-emotional processing of a traumatic event. This behavior is probably related to greater suffering but may be useful for the adaptation and growth of an individual in the future. Our findings highlight the importance of exploring not only positive but also negative aspects of psychological factors related to growth, which could lead to a better understanding of its development.

The importance of hope in the prediction of positive consequences of the COVID-19 pandemic was not significant. However, hope, in two factors (dispositional hope in the path component and perceived hope), was identified as a significant predictor of negative changes related to the COVID-19 pandemic. Our results indicate that when a person who has a goal and a plan to achieve it faces some obstacles (Path component) and at the same time believes that the current situation and difficulties can be controlled and overcome (perceived hope), positive beliefs can reduce negative psychological changes (such as the worsening of social relationships, loss of self-confidence, and lacking the meaning of life) during the pandemic. The importance of dispositional hope in the Agency component in the prediction of negative changes was not significant. Agency represents a significant component of dispositional hope, and it includes self-confidence in achieving defined goals. In our research, the concept of perceived hope appears to be a more significant component of self-confidence in achieving goals during the pandemic period. Perceived hope is understood as a deep trust for a positive outcome, especially in difficult life situations that are beyond our direct control and in deeper resources for coping built not only on trust in one's own strength but also on faith in something or someone beyond us (Krafft and Walker, 2018). Due to the COVID-19 pandemic, people were exposed to various unpredictable stressful life events for a long time, quite often without being able to control them. We assume that in such situations, people relied not only on their own strengths but also on self-transcendent sources in the process of achieving goals. Evidence that hope had a direct impact on improved mental health during the COVID-19 pandemic has been supported by several studies (Yildirim and Arslan, 2020, 2022; Gallagher et al., 2021; Genc and Arslan, 2021; Wider et al., 2022). Hopeful people are more creative and remain resolute in pursuit of their goals, which can lead to reduced levels of mental health disorders such as anxiety and depression (Arnau et al., 2007), practice more adaptive coping strategies in managing adverse life circumstances (Folkman, 2013), are better able to respond difficult situations, are more likely to be capable of bouncing back from a stressful situation and motivated toward goals, and create pathways for attaining a desired goal (Yildirim and Arslan, 2022). Other studies from the pandemic period found that hopelessness and desperation have been associated with negative outcomes, including suicidal ideation (Thakur and Jain, 2020).

The importance of life orientation (optimism and pessimism) in the prediction of negative changes was not significant. This finding can be explained by the different theoretical backgrounds of the hope and optimism concepts. Wider et al. (2022) state that optimists may believe that things will turn out the way they want to but may not possess the pathways to pursue goals related to what they hope to achieve, whereas hope focuses directly on the personal attainment of pursued goals and one's beliefs in their capability to achieve those goals. As we have already mentioned, dispositional hope manifests itself mainly in situations that are more personally relevant and are associated with specific goals. During the COVID-19 pandemic, many people experienced feelings of fear or being under threat. These situations required faith in their own abilities to find effective ways to achieve goals, thus decreasing the risk of negative psychological changes.

Last but not least, the different roles of dispositional hope and optimism with an impact on various life changes supports the perspective (Yang et al., 2014; Rand, 2018) that hope and optimism are similar but basically different constructs because they can predict various mental health-related consequences. Rand (2018) explains this perspective through the role of different coping strategies as mediating factors. Optimism should be negatively related to dysfunctional coping, while hope should be associated more with coping strategies related to the achievement of goals, such as active problem-focused coping.

## Limitations

This study has several limitations. First, the research sample size and the predominantly online data collection limited the desired representativeness of the research file and the possibility to formulate general conclusions. We are fully aware that through online surveys, only a selected group could have access to the study, possibly depending on their educational level or economic status. This approach was chosen due to the public measures related to the pandemic situation at the time of data collection. Regarding the sample size, multiple linear regression analysis (Enter method) was used for the analysis of models to predict the positive and negative changes. Power analysis was conducted using G^*^Power version 3.1.9.4 (Faul et al., 2007). The results for multiple linear regression with six predictors indicated the required sample size to achieve 80% power for detecting a medium effect, at a significance criterion of α = 0.05, *n* = 98. The obtained sample size of *n* = 102 is, from this aspect, sufficient for testing the hypotheses of the study.

Second, data on the occurrence of positive and negative changes in connection with the pandemic were collected during the fourth wave of the COVID-19 pandemic, 2 years after its outbreak. It is questionable whether the changes reported by the participants in the CiOQ-S questionnaire during the measurement period resulted from the current pandemic situation, whether their ability to evaluate was weakened, or the reported changes were the result of other situations. Despite these considerations, we assume that the pandemic, even in this period, had the potential to evoke changes in individuals' views of their lives. The course of the pandemic turned out to be a dynamic process. Similarly, at the time of data collection, when the onset of the fourth wave of the pandemic was reported and the omicron variant became the dominant circulating variant, people in Slovakia were exposed to diverse information, according to which, e.g., “*this is a less severe variant than delta variant, but on the other hand, extremely high numbers of infected people are expected”*. We assume that the aforementioned types of publicized information had the potential to trigger another wave of stress in people, the “*coronavirus fear”*, and to intensify uncertainty and anxiety.

Third, the approach used in this study does not clarify whether the obtained results support growth as a real, transformative phenomenon, or only as a product of a positive illusion. For this purpose, we propose to supplement the measurement of the presence of personal growth by, for example, the administration of a behavioral checklist with the inclusion of negative changes in the level of mental health, on a personal, emotional, and social level, and in activities of daily life. At the same time, we also see an opportunity for longitudinal research that would monitor the stability of personal growth.

Fourth, in the submitted research, one of our goals was to monitor the coincidence of the occurrence of positive and negative psychological changes in adults during the fourth wave of the COVID-19 pandemic, but we did not explicitly investigate how our participants experienced the pandemic due to their level of stress (in several studies, the inclusion of this variable has been shown to be beneficial for a deeper understanding of the psychological mechanisms associated with personality growth, e.g., Hu et al., 2021, Asmundson et al., 2021, and others).

## Conclusion

Despite the aforementioned limitations, we have assessed the submitted study from two main perspectives. First, we conducted parallel monitoring to track both positive and negative changes associated with the COVID-19 pandemic. This approach allowed us to gain a more comprehensive understanding of the potential shifts in participants' experiences related to the pandemic. Additionally, it prompted us to question whether these changes were genuine or merely perceived. Second, we incorporated two different orientations (optimism and pessimism) and two types of hope (dispositional and perceived) in regression models. These variables were utilized to predict the occurrence of positive and negative changes in relation to the COVID-19 pandemic. Our results indicated that life orientation and hope should be perceived as important resources to adapt to adverse life events during the COVID-19 pandemic, mainly in terms of supporting positive development and reducing the occurrence of developing negative psychological life changes.

Hope and optimism are promising targets for interventions to foster resilience during the COVID-19 pandemic, considering their potential link with positive expectations and adaptive responses. We recommend incorporating hope interventions into mental health support to enable individuals to develop the capacity to set and achieve personal goals and effectively utilize hope during times of stress or when facing life satisfaction challenges. Such interventions can be integrated into various forms of psychotherapy. To ensure the effectiveness of hope-based interventions, it is essential to validate them through randomized control trials (RCTs), comparing them with established gold-standard treatments and assessing optimism interventions. Several interventions focusing on fostering positive expectations, including hope (Cheavens and Guter, 2018) and optimism (Malouff and Schutte, 2017), have already been developed. Additionally, interventions targeting the most vulnerable individuals during the COVID-19 pandemic have been implemented, such as an RCT study of an internet-based positive psychology intervention for healthcare students in Tunisia, which resulted in increased levels of hope and optimism (Krifa et al., 2022). To determine the real effectiveness of optimism and hope in the process of coping with adverse life events, more evidence and studies are needed, focusing on better insight into the mechanism between positive psychological states and life changes in times of crisis. As we have already mentioned, the process of growth takes more time, the shift from negative to positive thinking occurs gradually, and both hope and optimism require some time to affect personal growth.

It has been demonstrated that integrating a comprehensive biocentric and psychological approach to disaster management, such as Body–Mind Interventions, Biodanza, and Ethical ecological strategies, could enhance people's preparedness and ability to cope with a pandemic, post-pandemic period, or crisis. Pandemics tend to disrupt connections and cause dissociations across various levels, including the immune system, hormonal system, central nervous system, instincts and behavior, motor activity and sympathetic arousal, desire for connection and physical separation, and lack of contact and physicality, as well as the mind–body connection and the interplay between feeling, thinking, and acting. Based on biocentrically oriented studies by Parker et al. (2020) and Stueck (2021), we consider that working with the body through practices such as practicing meditation and autogenic training plays a crucial role in stress regulation, reducing the secretion of stress hormones such as adrenocorticotropic hormone (ACTH), cortisol, and catecholamines. Moreover, these practices have a positive impact on various aspects of mental health by fostering positive expectations, hope, and optimism, which can contribute to positive psychological changes related to the pandemic.

## Data availability statement

The raw data supporting the conclusions of this article will be made available by the authors, without undue reservation.

## Ethics statement

Ethical review and approval was not required for the study on human participants in accordance with the local legislation and institutional requirements. The patients/participants provided their written informed consent to participate in this study.

## Author contributions

EJ and LP conducted the literature review and the analysis, wrote the first draft of the manuscript, conceptualized the study design, and conducted the study. MR, LR, and TS assisted with the analysis and commented on drafts. MR and LP conducted the literature review. LP collected the data. All authors have reviewed and approved the final manuscript.
